# Resistance, mechanism, and fitness cost of specific bacteriophages for *Pseudomonas aeruginosa*

**DOI:** 10.1128/msphere.00553-23

**Published:** 2024-02-01

**Authors:** Luozhu Feng, Huanchang Chen, Changrui Qian, Yining Zhao, Weixiang Wang, Yan Liu, Mengxin Xu, Jianming Cao, Tieli Zhou, Qing Wu

**Affiliations:** 1 Department of Clinical Laboratory, Key Laboratory of Clinical Laboratory Diagnosis and Translational Research of Zhejiang Province, the First Affiliated Hospital of Wenzhou Medical University, Wenzhou, Zhejiang Province, China; 2 Department of Clinical Laboratory, the First Affiliated Hospital of Ningbo University, Ningbo, Zhejiang Province, China; 3 Department of Medical Lab Science, School of Laboratory Medicine and Life Science, Wenzhou Medical University, Wenzhou, Zhejiang Province, China; University of Rochester, Rochester, New York, USA

**Keywords:** *Pseudomonas aeruginosa*, bacteriophage, resistance, mechanism, cost of fitness

## Abstract

The bacteriophage is an effective adjunct to existing antibiotic therapy; however, in the course of bacteriophage therapy, host bacteria will develop resistance to bacteriophages, thus affecting the efficacy. Therefore, it is important to describe how bacteria evade bacteriophage attack and the consequences of the biological changes that accompany the development of bacteriophage resistance before the bacteriophage is applied. The specific bacteriophage vB3530 of *Pseudomonas aeruginosa* (*P. aeruginosa*) has stable biological characteristics, short incubation period, strong *in vitro* cleavage ability, and absence of virulence or resistance genes. Ten bacteriophage-resistant strains (TL3780-R) were induced using the secondary infection approach, and the plaque assay showed that vB3530 was less sensitive to TL3780-R. Identification of bacteriophage adsorption receptors showed that the bacterial surface polysaccharide was probably the adsorption receptor of vB3530. In contrast to the TL3780 parental strain, TL3780-R is characterized by the absence of long lipopolysaccharide chains, which may be caused by base insertion of *wzy* or deletion of *galU*. It is also intriguing to observe that, in comparison to the parent strain, the bacteriophage-resistant strains TL3780-R mostly exhibited a large cost of fitness (growth rate, biofilm formation, motility, and ability to produce enhanced pyocyanin). In addition, TL3780-R9 showed increased susceptibility to aminoglycosides and chlorhexidine, which may be connected to the loss and down-regulation of *mexX* expression. Consequently, these findings fully depicted the resistance mechanism of *P. aeruginosa* to vB3530 and the fitness cost of bacteriophage resistance, laying a foundation for further application of bacteriophage therapy.

**IMPORTANCE:**

The bacteriophage is an effective adjunct to existing antibiotic therapy; However, bacteria also develop defensive mechanisms against bacteriophage attack. Thus, there is an urgent need to deeply understand the resistance mechanism of bacteria to bacteriophages and the fitness cost of bacteriophage resistance so as to lay the foundation for subsequent application of the phage. In this study, a specific bacteriophage vB3530 of *P. aeruginosa* had stable biological characteristics, short incubation period, strong *in vitro* cleavage ability, and absence of virulence or resistance genes. In addition, we found that *P. aeruginosa* may lead to phage resistance due to the deletion of *galU* and the base insertion of *wzy*, involved in the synthesis of lipopolysaccharides. Simultaneously, we showed the association with the biological state of the bacteria after bacteria acquire bacteriophage resistance, which is extremely relevant to guide the future application of therapeutic bacteriophages.

## INTRODUCTION

*Pseudomonas aeruginosa* (*P. aeruginosa*) is a significant pathogen in hospital- and community-acquired infections, which can cause wound infections, bacteremia, respiratory tract infections, and urinary tract infections (1, 2). In addition, *P. aeruginosa* produces a variety of virulence factors, including rhamnolipid, pyocyanin, and biofilm, and shows high resistance to a series of commonly used antibiotics, including β-lactams, fluoroquinolones, and aminoglycosides, which leads to high morbidity and mortality (3, 4). According to reports from the U.S. Centers for Disease Control and Prevention, it is estimated that there are approximately 51,000 healthcare-associated infections caused by *P. aeruginosa* each year in the United States. Thirteen percent of *P. aeruginosa* are multidrug-resistant (MDR), approximately 400 people die each year from this type of infection (5, 6), and *P. aeruginosa* is listed in the “critical” category of the World Health Organization’s (WHO) priority list of bacterial pathogens. Therefore, the treatment of *P. aeruginosa* infection is a great clinical challenge. Carbapenems are the final line of treatment for *P. aeruginosa* infections (7). However, due to the extensive use of carbapenem, the isolation rate of carbapenem*-*resistant *P. aeruginosa* is rising year over year, posing significant difficulties for clinical anti-infection treatment (8).

Bacteriophages, a type of specialized bactericidal virus, are an effective adjunct to existing antibiotic therapy. Although they were found around a century ago and were first used to treat human infections in 1919, there have been many successful applications of bacteriophages in the treatment of infections, including bone graft microbial infection (4), necrotizing pancreatitis complicated by microbial infection (9), and burn wound infection (10). However, with the interplay between bacteria and phages, the host bacteria will eventually evolve resistance to phages, thus affecting the efficacy of bacteriophages. Bacteria have a number of defense mechanisms against bacteriophages, including the ability to prevent bacteriophage adsorption on their surface, specifically cleave nucleic acids injected into them using the restriction–modification (R-M) system and CRISPR/Cas system, and even induce the suicide of bacteriophage-infected cells using the abortive infection (Abi) system (11). Blocking bacteriophage adsorption seems to be the most common strategy for bacteria to defend against bacteriophage infection. Shayla Hesse et al. found that multidrug-resistant *Klebsiella pneumoniae* weakened its adsorption by phages through multiple mutations (12). Transposable element insertions into the *Escherichia coli* polysialic acid gene cluster result in resistance to the K1F bacteriophage (13). However, the resistance mechanism of *P. aeruginosa* to different bacteriophages may be different, and whether there are other mechanisms leading to resistance of *P. aeruginosa* to bacteriophages still needs to be further explored. Interestingly, it has also been found that in some cases, bacteriophage-resistant bacteria lose other adaptive advantages (i.e., the evolution of a favorable trait at the same time as the performance of another trait). It has been reported that bacteriophage-induced resistance to bacteria leads to slower growth, decreased virulence, and increased susceptibility to various antimicrobial agents (14). Bacteriophage-resistant bacteria are more susceptible to host-clearance mechanisms by the immune system (the complement system and phagocytosis, etc.) (15). However, the fitness cost has been shown to vary across species and genera of bacteria (16–20), so it is crucial to describe how bacteria evade bacteriophage attack and the accompanying physiological change outcomes after bacteria develop bacteriophage resistance before bacteriophage application. In conclusion, the purpose of this study is to identify specific bacteriophages with potent lysis activity against carbapenem-resistant *P. aeruginosa*, investigate their biological and genomic characteristics, and explore potential mechanisms underlying bacteriophage resistance in *P. aeruginosa* as well as potential fitness costs.

## RESULTS

### Characterization of *P. aeruginosa* specific bacteriophage vB3530

**Fig 1:**
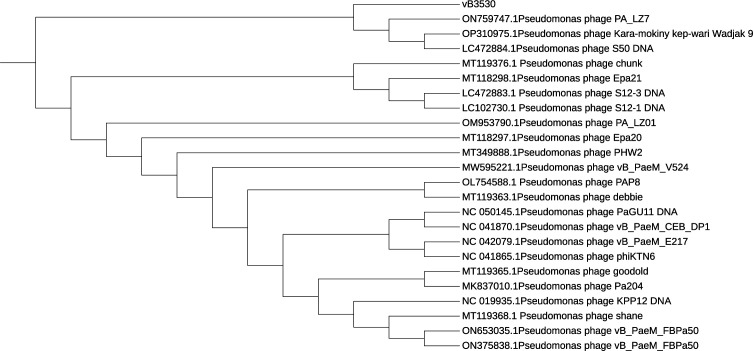
Phylogenetic tree constructed based on the whole-genome of bacteriophage vB3530.

**Fig 2:**
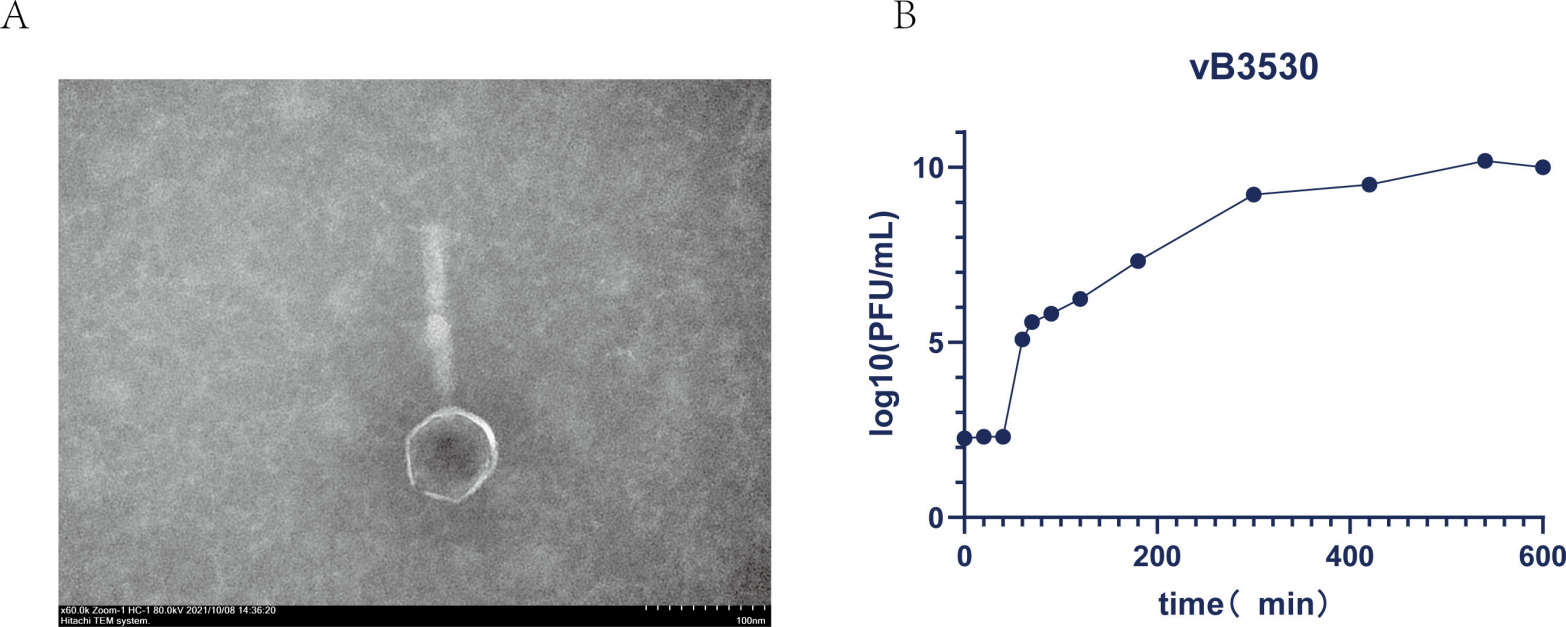
(A) Transmission electron microscopy of bacteriophage vB3530. (B) One-step growth curve of bacteriophage vB3530.

In our previous study, we isolated the lytic bacteriophage vB3530 of *P. aeruginosa* from the sewage treatment plant at the Center of the First Affiliated Hospital of Wenzhou Medical University. Through whole-genome sequencing analysis and transmission electron microscopy observation, it was found that vB3530 belonged to the *Pbunavirus* genus of the Caudoviricetes class (21, 22). In order to understand the homology relationship between bacteriophage vB3530 and other bacteriophages, the phylogenetic evolution of bacteriophage vB3530 was analyzed based on the whole genome, and the results are shown in Fig. 1, which showed that bacteriophage vB3530 was closely related to bacteriophage PA_LZ7, Kara-mokiny kep-wari Wadjak 9 (23), and S50. At the same time, 16 strains of *P. aeruginosa* with a definite specimen source and sequence type were selected as the test strains for the host spectrum experiment, and the results showed that bacteriophage vB3530 was positive for 13/16 strains of *P. aeruginosa* (Table S1). Among them, *P. aeruginosa* TL3780 was isolated from urine samples of emergency care patients, which had an MIC of 16 µg/mL for imipenem and 32 µg/mL for ertapenem. Also, bacteriophage vB3530 also had lytic capacity against carbapenem-resistant *P. aeruginosa* TL3780. The diameter of the plaque formed by the vB3530 against TL3780 was approximately 1 mm according to the double-layer agar plate method, and the optimum multiplicity of infection (MOI) was 0.01. According to the one-step growth curve, the latent time for bacteriophage vB3530 was approximately 40 min, the lysis time was around 500 min, and the burst size was approximately 300 bacteriophages per infected cell (Fig. 2).

### Resistance of bacteriophage-resistant *P. aeruginosa* TL3780 to specific bacteriophages and its mechanism

**Fig 3:**
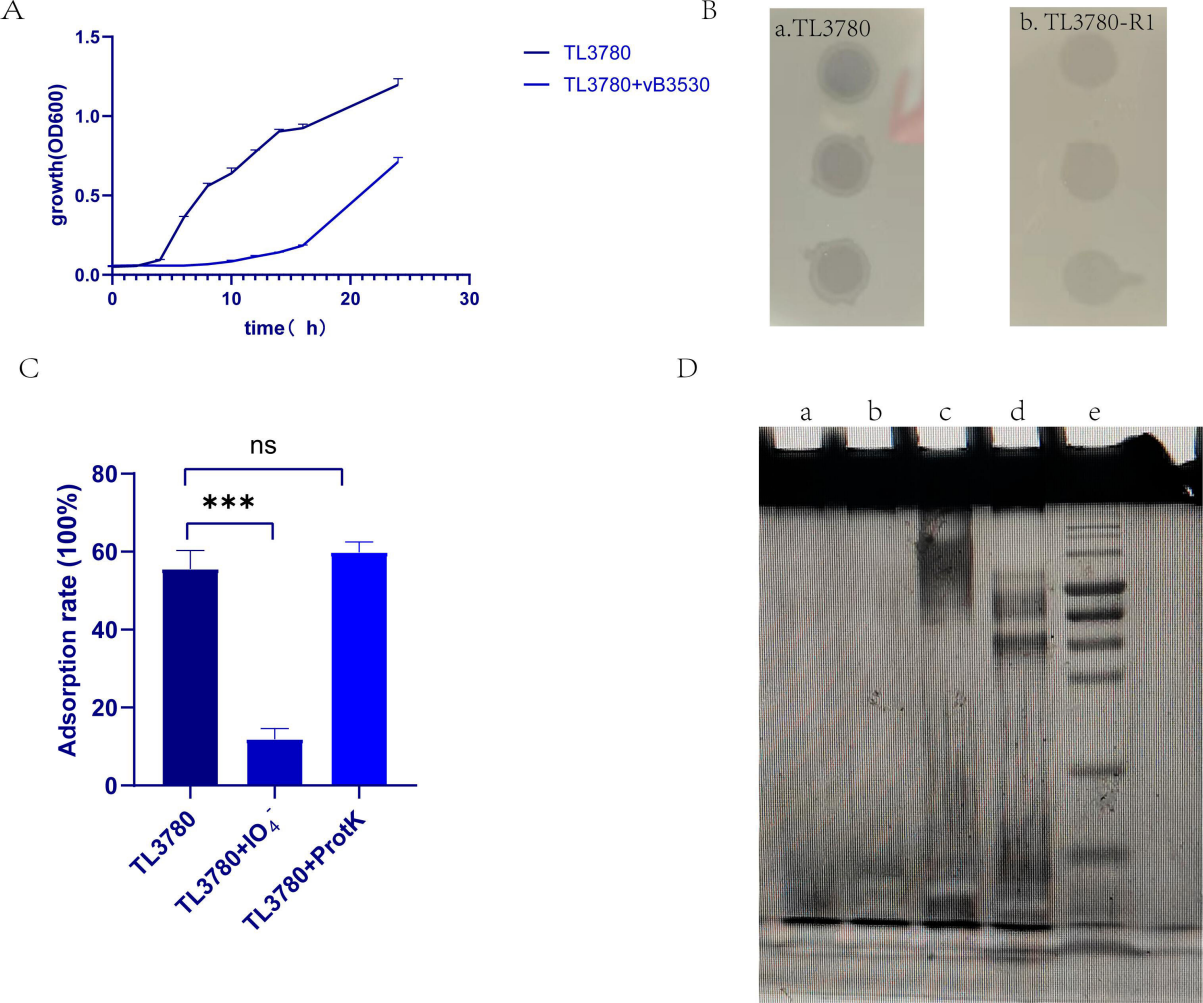
Resistance mechanism of TL3780 to bacteriophage vB3530. (A) The co-incubation process of bacteriophages vB3530 and TL3780; (B) Sensitivity of TL3780 parental strain and TL3780 resistant strains to bacteriophage vB3530; (C) Identification of phage adsorption receptors. IO_4_^-^, treated with periodate; ProtK, treated with protease K. (D) Lipopolysaccharide (LPS) correlation between TL3780 and phage-resistant strain TL3780-R was detected by SDS-PAGE. a, SDS-PAGE results of the phage-resistant strain TL3780-R1’s LPS; b, SDS-PAGE results of the phage-resistant strain TL3780-R2’s LPS; c, SDS-PAGE results of the phage-resistant strain TL3780-R9’s LPS; d, SDS-PAGE results of the parental strain TL3780’s LPS; e, marker.

To investigate the bacteriophage resistance mechanism, *P. aeruginosa* TL3780 was co-cultured with bacteriophage vB3530. After bacteriophages were co-cultured with *P. aeruginosa* for 14 hours, bacteriophage-resistant *P. aeruginosa* was detected, and we randomly isolated 10 resistant strains (TL3780-R1 to R10) at 24 h (Fig. 3A). The bacterial growth curve and the results of plaque assay revealed lower lysis ability of vB3530 to these bacteriophage-resistant strains (Fig. 3B). Two types of phage-resistant strains were isolated: one was pyocyanin-producing phage-resistant strain (TL3780R1-R8; TL3780-R10) and the other was a phage-resistant strain producing a reddish-brown pigment (TL3780-R9). In order to explore the type of the bacteriophage adsorption receptor, we treated *P. aeruginosa* TL3780 with periodate or protease K to destroy its surface polysaccharide or protein structure and then detected the adsorption rate of bacteriophage vB3530. The results showed that the bacteriophage adsorption rate of bacteria treated with periodate decreased significantly, while the protease K treatment did not affect the adsorption efficiency of bacteriophage to TL3780, suggesting that the bacterial surface polysaccharide is likely to be the adsorption receptor of bacteriophage vB3530 to *P. aeruginosa*, which plays an important role in bacteriophage infection (Fig. 3C) (24, 25).

**Fig 4:**
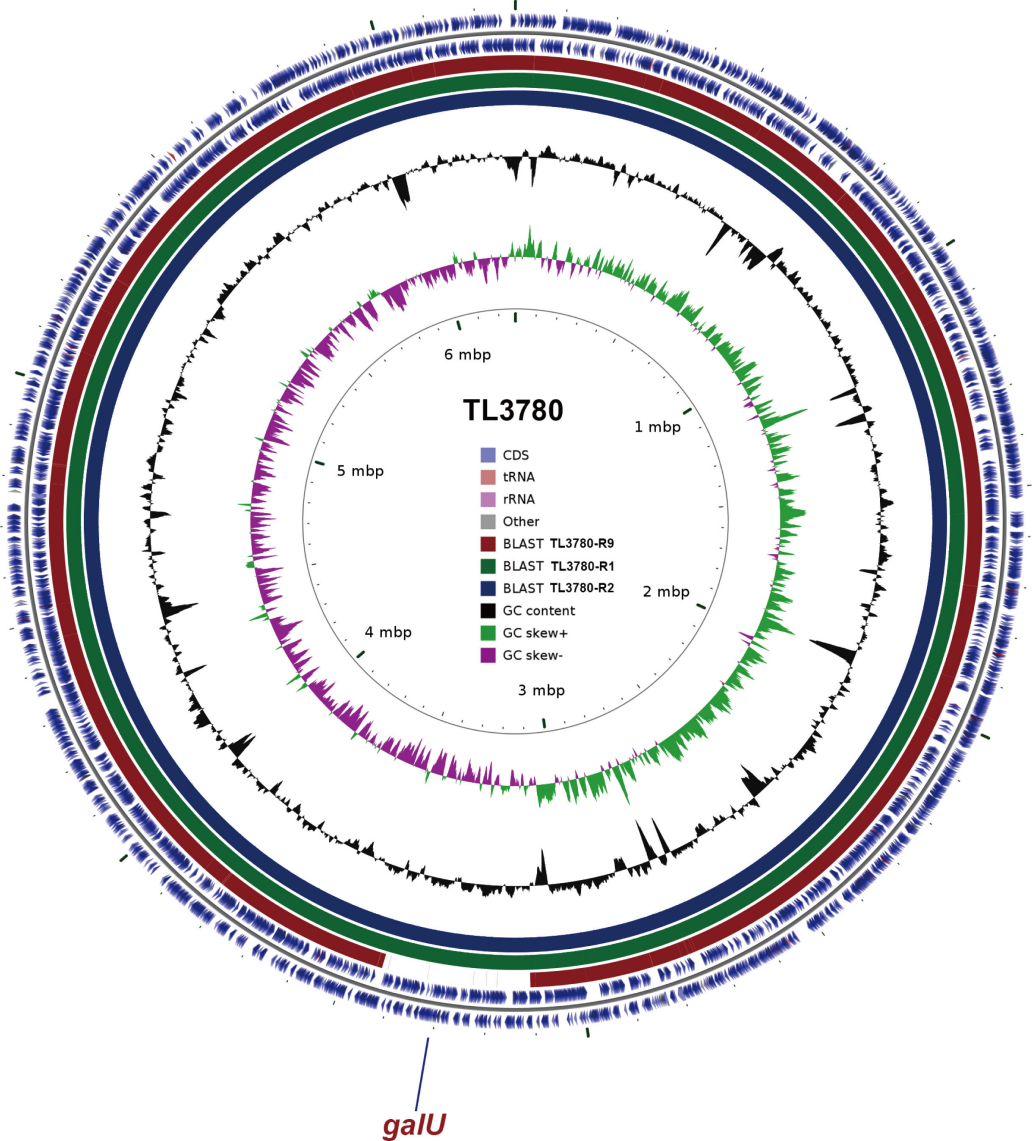
Genomic maps of TL3780 parental strains and phage-resistant strains (TL3780-R1, TL3780-R2, and TL3780-R9).

**Fig 5:**
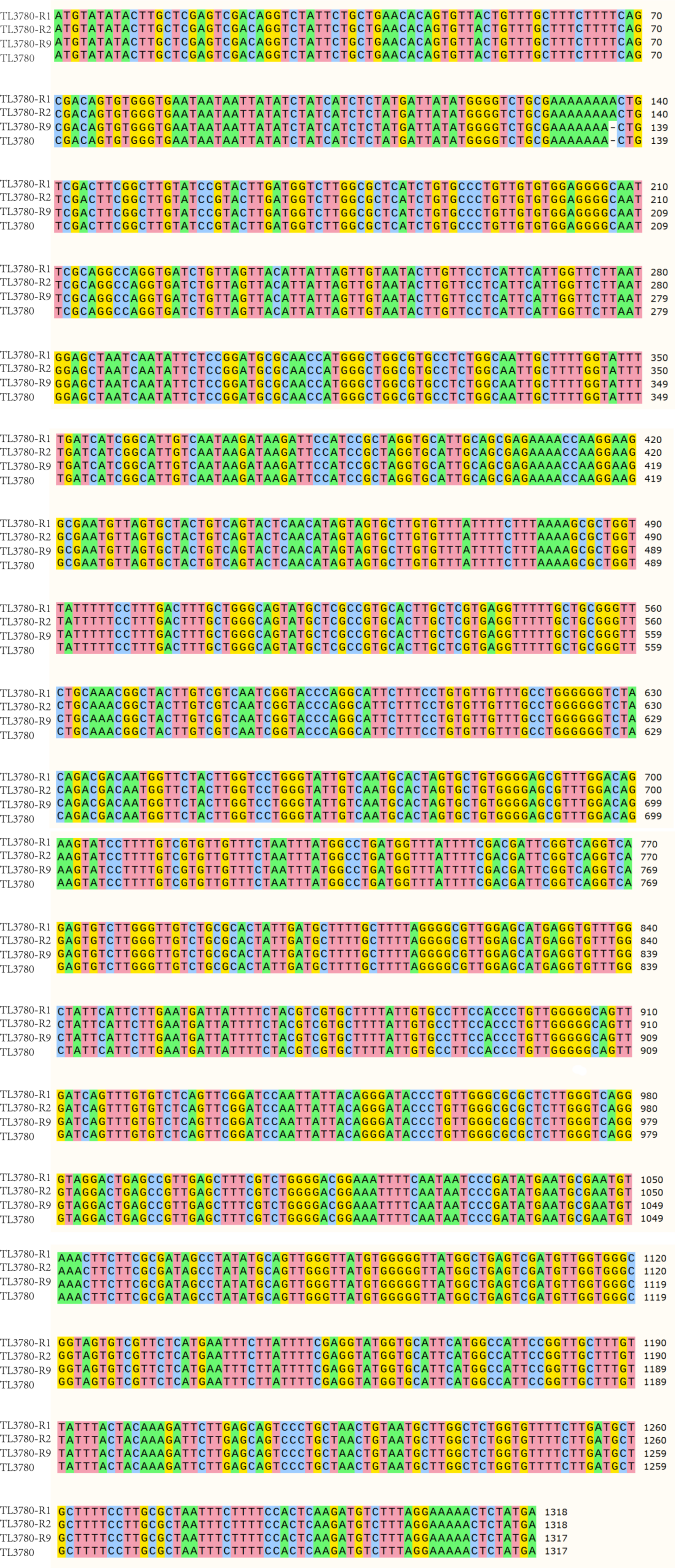
Multiple sequence alignment of the *wzy* gene between TL3780 parental strain and phage-resistant strains (TL3780-R1, TL3780-R2, and TL3780-R9).

Lipopolysaccharide (LPS) is the main surface polysaccharide of *P. aeruginosa*, which is composed of lipids and polysaccharides and is a component of the outer cell membrane (26). To understand the LPS composition of *P. aeruginosa* TL3780 and bacteriophage-resistant *P. aeruginosa* TL3780-R, the LPSs of *P. aeruginosa* TL3780 and bacteriophage-resistant *P. aeruginosa* TL3780-R1, TL3780-R2, and TL3780-R9 were extracted. The result showed that TL3780-R1, TL3780-R2, and TL3780-R9 had a long-chain deletion of LPS, as demonstrated by SDS-PAGE silver staining (Fig. 3D). As a result, we surmised that LPS loss may cause TL3780 to become resistant to vB3530. The parental strain TL3780 and three vB3530-resistant strains (TL3780-R1, TL3780-R2, and TL3780-R9) were then chosen for whole-genome sequencing (WGS). WGS results showed that there were large gene deletion fragments in the TL3780-R9 genome, including 290 genes, such as the *galU* gene related to LPS synthesis, the *hmgA* gene related to pigmentation synthase, and *mexX* and *mexY* genes related to efflux pump (Fig. 4). GalU is a UDP-glucose glucophosphorylase responsible for synthesis of UDP-glucose from glucose 1-phosphate and UTP, and mutations in *galU* cause LPS in *Aeromonas hydrophila* to lack the O antigen (27). Compared with the parental strains, TL3780-R1 and TL3780-R2 had base insertion 136 insA in the *wzy* gene (Fig. 5), and the *wzy* gene encoded O antigen polymerase Wzy, which played an important role in the synthesis of bacterial LPS (28). Therefore, it is further speculated that TL3780 can cause LPS synthesis dysfunction through the deletion of LPS synthesis-related gene *galU* and *wzy* base insertion, which leads to decreased sensitivity to phages.

### Fitness cost of bacteriophage-resistant *P. aeruginosa* TL3780

**Fig 6:**
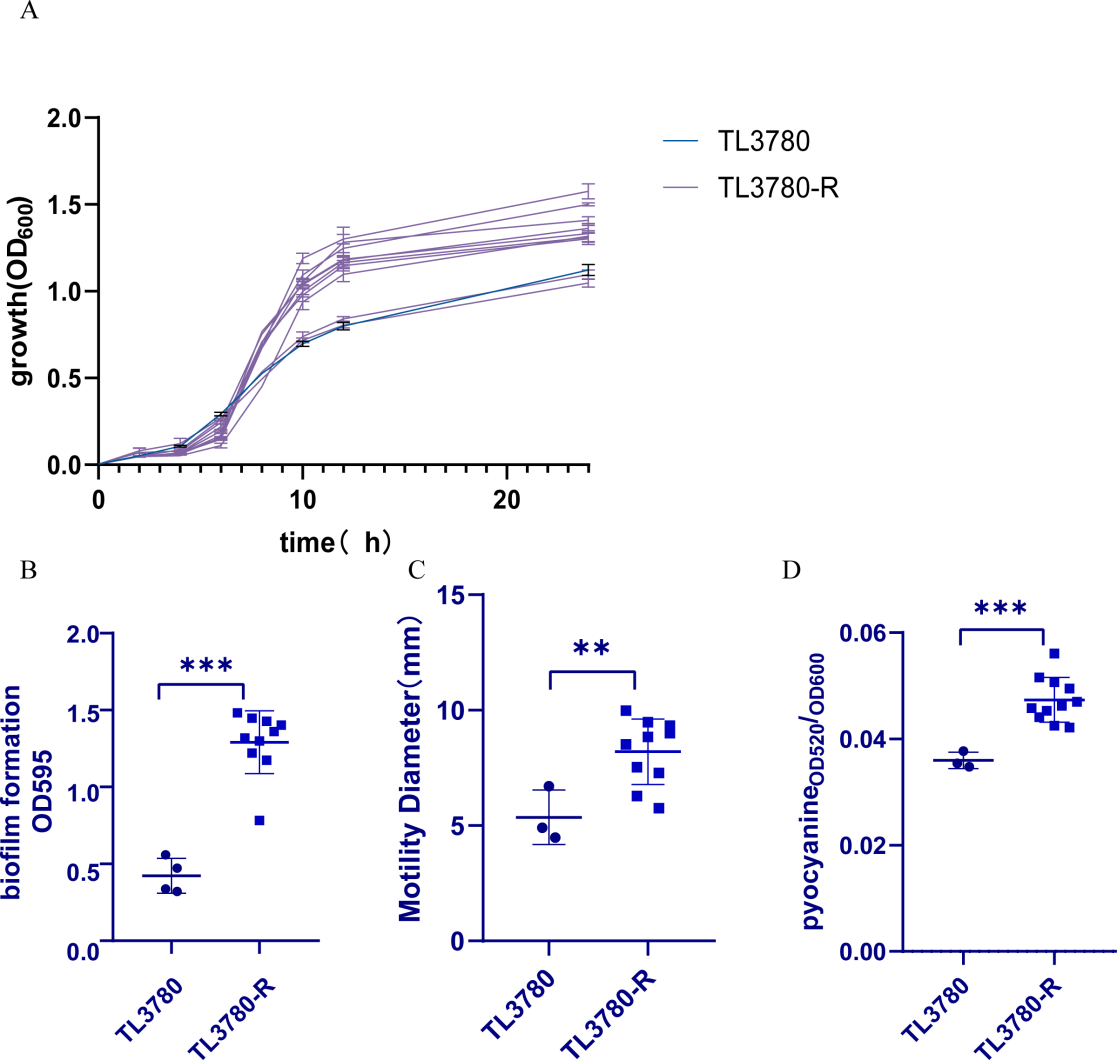
Fitness cost of TL3780 parental strain and phage-resistant strain TL3780-R. (A) Growth of TL3780 parental strain and phage-resistant strain TL3780-R. (B) Biofilm formation of TL3780 parental strain and phage-resistant strain TL3780-R. (C) Motility of TL3780 parental strain and phage-resistant strain TL3780-R. (D) Production of pyocyanin between TL3780 parental strain and phage-resistant strain TL3780-R.

**Fig 7:**
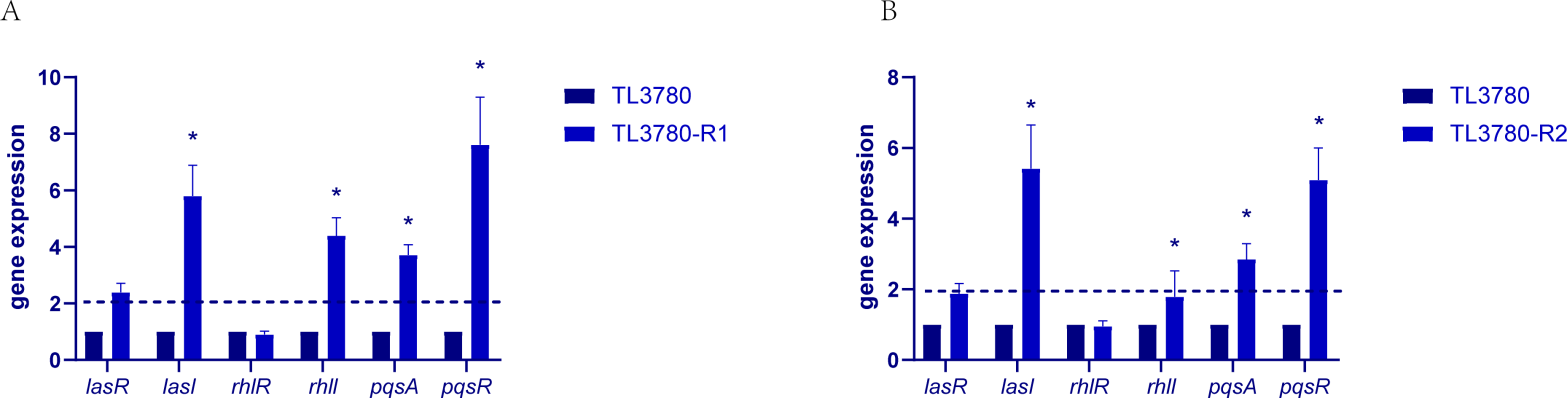
QS gene expression of TL3780 and phage-resistant TL3780-R1 and TL3780-R2 strains were detected by RT-qPCR. (A) QS gene expression of TL3780 parental strain and phage-resistant strain TL3780-R1. (B) QS gene expression of TL3780 parental strain and phage-resistant strain TL3780-R2.

Interestingly, it has been found that in some cases, bacteriophage-resistant bacteria lose other adaptive advantages. However, fitness costs have been shown to vary across species and genera of bacteria (17–20). We tested the changes of fitness cost between resistant strain TL3780-R and the parental strain TL3780, including growth rate, biofilm formation ability, motility ability, and pyocyanin production ability (Fig. 6). The results showed that the resistant strain TL3780-R grew faster than its parental strain. The crystal violet staining test revealed that the majority of resistant strains demonstrated improved biofilm-forming capacity. The motility test and pyocyanin test findings revealed that the majority of resistant strains had improved motility and pyocyanin production capacity. The quorum-sensing (QS) system is able to regulate growth rate, biofilm, motility, and pyocyanin synthesis (29). Consequently, we detected the QS-related gene (*lasR*, *lasl*, *rhlR*, *rhll*, *pqsA,* and *pqsR*) expression of TL3780-R1 and TL3780-R2. The QS gene expression of TL3780-R1 and TL3780-R2 was up-regulated in comparison to that of TL3780, according to the results of quantitative reverse transcription PCR (RT-qPCR) (Fig. 7). Therefore, it is hypothesized that the upregulation of QS gene expression in phage-resistant *Pseudomonas aeruginosa* mutants may be responsible for the changes in growth rate, biofilm formation, motility, and pyocyanin production.

### Changes in antimicrobial susceptibility of bacteriophage-resistant *P. aeruginosa* TL3780

**Fig 8:**
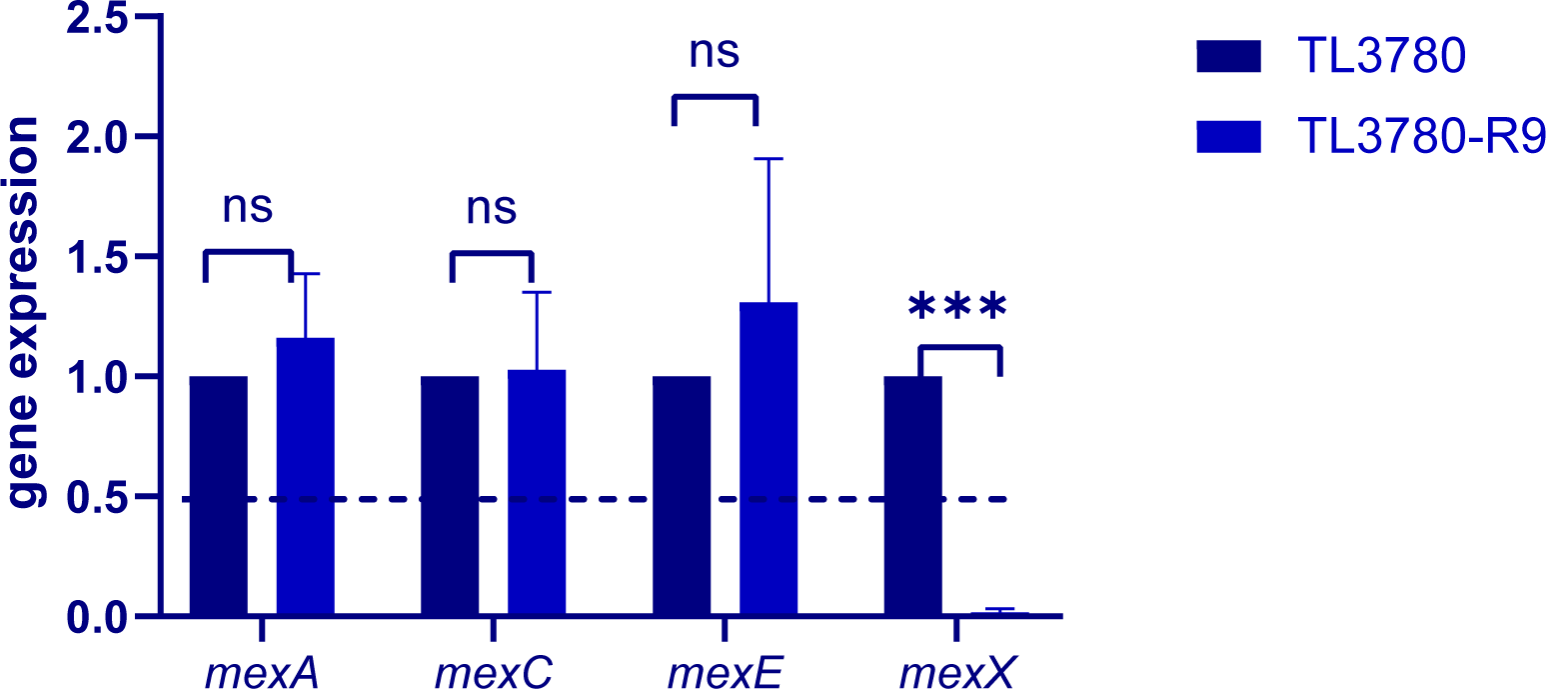
Efflux pump gene expression of TL3780 and phage-resistant strain TL3780-R9 was detected by RT-qPCR.

Next, we compared the parental strain TL3780 and the bacteriophage-resistant strains (TL3780-R1 to TL3780-R10) or antimicrobial susceptibility. It is intriguing to note that only producing the reddish-brown pigment TL3780-R9 has shown enhanced susceptibility to chlorhexidine and aminoglycosides (gentamicin, amickacin, and tobramycin), with the minimum-inhibitory concentration (MIC) of chlorhexidine reduced from 32 µg/mL to 2 µg/mL and the MICs of gentamicin, amikacin, and tobramycin reduced from 4, 4, and 1 to 0.25, 0.5, and 0.125 µg/mL, respectively (Table 1). Comparing the WGS data of TL3780-R9 to that of TL3780-R1, TL3780-R2, and TL3780 parental strains, we discovered that more gene deletions, such as the *mexX*, *hmgA*, and *galU,* are present in TL3780-R9 (Fig. 8). Of note, *mexX* is assumed to be involved in chlorhexidine and aminoglycoside sensitivity since it is an aminoglycoside-inducible efflux pump.

**TABLE 1:** Antimicrobial susceptibility results of TL3780 and its bacteriophage-resistant strains^*a*^

Isolate	MIC (μg/mL）											
	CHX	IPM	ETP	MEM	LVX	CIP	FEP	GEN	AMK	TOB	ATM	COL
Parental TL3780	32	16^R^	32^R^	4^I^	0.5	0.25	2	4	4	1	4	2^I^
TL3780-R1	32	16^R^	32^R^	4^I^	0.5	0.25	2	4	4	1	8	2^I^
TL3780-R2	32	16^R^	32^R^	4^I^	0.5	0.25	2	4	4	1	4	2^I^
TL3780-R3	32	16^R^	32^R^	4^I^	0.5	0.25	2	4	4	1	4	2^I^
TL3780-R4	16	16^R^	32^R^	4^I^	0.5	0.25	2	4	4	1	4	2^I^
TL3780-R5	16	16^R^	32^R^	4^I^	0.5	0.25	4	4	4	1	4	2^I^
TL3780-R6	16	16^R^	32^R^	4^I^	0.5	0.25	4	4	4	1	8	2^I^
TL3780-R7	16	16^R^	32^R^	4^I^	0.5	0.25	4	4	8	1	8	2^I^
TL3780-R8	32	16^R^	32^R^	4^I^	0.5	0.25	4	4	4	1	4	2^I^
TL3780-R9	2	16^R^	32^R^	4^I^	0.5	0.25	4	0.25	0.5	0.125	4	2^I^
TL3780-R10	32	16^R^	32^R^	4^I^	0.5	0.25	4	4	4	1	4	2^I^

^*a*^ CHX, chlorhexidine; IPM, imipenem; ETP, ertapenem; MEM, meropenem；LVX, levofloxacin; CIP, ciprofloxacin; FEP, cefepime; GEN, gentamicin; AMK, amikacin; TOB, tobramycin; ATM, aztreonam; COL, colistin.

## DISCUSSION

The bacteriophage as a supplemental antimicrobial therapy is considered to be one of the most efficient strategies for combating bacterial infection by scientists (30, 31). In June 2023, the European Parliament proposed a motion for solving antibiotic resistance by asking the member states of the Council of Europe (32). In this study, we explored the potential of bacteriophage vB3530 as a potential antimicrobial agent and possible challenges for future applications. Therapeutic bacteriophages need to have a well-defined lytic bactericidal effect and remove contaminated bacterial debris, in addition to determining the identity of the bacterial host receptor for any therapeutic phage, which will provide important information about the emergence of phage resistance, evolutionary tradeoffs, and use of combination therapies that are unlikely to produce a phage-resistant host (33). A one-step growth curve was used to examine the growth characteristics of bacteriophage vB3530, which helped us comprehend the bacteriophage’s capacity for lysis. According to the one-step growth curve, the latent time for bacteriophage vB3530 was approximately 40 min, the lysis time was around 500 min, and the burst size was approximately 300 bacteriophages per infected cell. The short latent period and large burst size are regarded as hallmarks of efficient bacteriophage lysis (34). For bacteriophage application, a clear genetic background is just as important as a strong lytic state. In our previous work, we had sequenced the whole genome of phage vB3530 and showed that phage vB3530 does not carry any resistance genes or virulence genes (34), indicating that vB3530 has the potential for biological and control medical treatment.

Despite the best clinical prevention efforts, clinically significant bacteriophage resistance will continue to emerge. Even in the case of Tom Patterson’s “successful” bacteriophage treatment, *Acinetobacter baumannii* isolates grown from patients 8 days after the start of bacteriophage treatment showed resistance to all eight bacteriophages in the initial cocktail (12). Therefore, understanding and clarifying the phage recognition receptors and the emergence of phage resistance will be of great help to the future application of phages. In this study, after treating bacteria with periodate and protease K, we identified that the recognition receptor of bacteriophage vB3530 may be a polysaccharide substance. In the subsequent process of phage and *P. aeruginosa* co-evolution, *P. aeruginosa* evolved resistance to phages, and, interestingly, we found that phage-resistant strains showed two forms, one was still producing pyocyanin and the other was producing a reddish-brown pigment. Both Keisuke Nakamura and Shen et al. discovered phage-resistant *P. aeruginosa* mutants with reddish-brown pigmentation during the co-evolution of *P. aeruginosa* and bacteriophages, which were mostly due to the loss of *hmgA* which encodes a homologous acid-metabolizing enzyme (35, 36). In order to further elucidate the resistance mechanism of phages, we selected two pyocyanin-producing phage-resistant strains (TL3780-R1 and TL3780-R2) and one phage-resistant strain producing a reddish-brown pigment (TL3780-R9) for SDS-PAGE and whole-genome sequencing. SDS-PAGE results showed that compared with parental strains, all three strains had long-chain deletion. Whole-genome sequencing results showed that the pyocyanin-producing phage-resistant strains had base insertion 136 insA in the *wzy* gene. While consistent with previous results, the phage-resistant strains producing reddish-brown pigment had deletion of large gene fragments, including the *hmgA*, *galU*, *mexX,* and *mexY* genes. GalU is a UDP-glucose pyrophosphorylase responsible for the synthesis of UDP-glucose from glucose 1-phosphate and UTP, and mutations in *galU* cause the LPS of *Aeromonas hydrophila* to lack the O antigen (27); the *wzy* gene encodes O antigen polymerase (Wzy), which plays an important role in the synthesis of bacterial LPS (37). Therefore, we hypothesized that TL3780 could be inhibited from LPS synthesis by the deletion of LPS synthesis-related gene *galU* and the insertion of *wzy* base, thus leading to its decreased sensitivity to phages. However, it has been reported that phages may have a variety of recognition receptors (primary and secondary receptors) (38), and we did not exclude the possibility of other recognition receptors of phages. MexX is thought as an aminoglycoside-inducible efflux pump (39–41), and the deletion of *mexX* may also be the reason for the increased sensitivity to produce the reddish-brown pigment by the bacteriophage-resistant strain TL3780-R9 to chlorhexidine and aminoglycosides (gentamicin, amikacin, and tobramycin).

Despite the existence of bacteriophage resistance, it sometimes comes with trade-offs of virulence and antimicrobial sensitivity, which are expected to improve treatment success (42). However, bacteriophage resistance is not always expensive, and it may be influenced by the genetic profile of the bacterium and the surrounding environment (43). The results of this study showed that compared with the parental strain, most of the resistant strain TL3780-R had accelerated growth rate, enhanced biofilm formation ability, enhanced motility, and enhanced pyocyanin production ability, including the two strains TL3780-R1 and TL3780-R2, which was different from the parallel evolution of bacteriophage resistance and virulence loss of *P. aeruginosa* in *in vitro* and *in vivo* bacteriophage treatment in other studies (43, 44). Since *P. aeruginosa* has a complex QS network, which may play an important role in its adaptation ability, regulating biofilm and pyocyanin production, motility, and other virulence factors (45), we selected these two strains TL3780-R1 and TL3780-R2 for subsequent QS gene expression detection. Xuan et al. also reported that AHL produced by the quorum-sensing system can regulate its resistance to phages, and AHL can reduce phage adsorption by down-regulating LPS expression, thus resisting phage infection (46). The results of RT-qPCR showed that compared with TL3780, the QS gene expression of TL3780-R1 and TL3780-R2 was up-regulated. These results provide a possible idea for the changes of growth rate, biofilm, motility, and pyocyanin production phenotype of bacteriophage-resistant *P. aeruginosa*.

Although the regular pattern of bacteriophage-resistant evolution in *P. aeruginosa* has been highlighted, it is also important to take into account its stochastic nature. Our genetic investigations are unable to fully explain the variety of resistance traits and adsorption deficiencies found in bacteriophage-resistant mutants from co-cultures (47). Although this level of phenotypic variation would not be clinically meaningful on its own, it evolves under extremely consistent laboratory settings as significant. In order to evaluate whether or how these data might be used to direct future therapeutic bacteriophage selection, further characterizations of bacteriophage resistance development *in vitro* and *in vivo* are required.

## MATERIALS AND METHODS

### Bacterial strains and bacteriophage

A total of 16 strains of *P. aeruginosa* isolated from various types of specimens sent to the clinical inpatients of the First Affiliated Hospital of Wenzhou Medical University from 2017 to 2018 were collected for phage host profiling, and the duplicate strains isolated from the same site of the same patient were excluded. Carbapenem-resistant *P. aeruginosa* TL3780, which can be lysed by phage vB3530, was used as the experimental strain for phage–bacterial co-evolution, and 10 phage-resistant strains (TL3780-R1 to TL3780-R10) were isolated for the subsequent fitness cost analysis. The bacteriophages vB3530 used in this study were obtained from our team’s previous research and were isolated from the sewage plant from the First Affiliated Hospital of Wenzhou Medical University (22).

### Host spectrum of the bacteriophage

The tested strains were obtained from 16 strains of *P. aeruginosa* from the previous research in our laboratory. Hundred microliters of bacterial solution of logarithmic phase were evenly mixed with 8 mL of semi-solid agar at a temperature of about 60°C, and then the abovementioned mixture was evenly spread on the LB agar. When the semi-solid agar was slightly coagulated and was in a semi-flowing state, 5 µL of the phage lysate (about 10^10^ PFU/mL) was added to the surface of the bacteria-containing semi-solid agar. After the phage lysate was fully absorbed, the plate was placed in a constant temperature incubator at 37°C, and the plaque formed by the phage was observed on the plate 6–10 h later. If the plaque is present, the host spectrum test is positive; if not, it is negative (48).

### One-step growth curves

The optimal MOI and one-step growth curves were performed as described by Leuschner et al. and Pajunen et al. with some modifications (49). Briefly, phages were added to 5 mL of the log-phase TL3780 culture (10^8^ CFU/mL) to achieve an MOI of 10, 1, 0.1, 0.01, 0.001, or 0.0001 and then incubated at 37 °C, 220 rpm for 4 h. The culture supernatant was then filtered through a 0.22-mm filter, and the titer of the phage in the supernatant was measured using a double-layer agar plate method. Three replicates were conducted for determination. The MOI resulting in the highest phage titer was considered the optimal MOI of the bacteriophage (49).

The one-step growth curve of a bacteriophage reflects dynamic changes in the number of particles during bacteriophage replication. Briefly, 30 mL of an early-exponential-phase culture (OD_650_ = 0.1–0.2) was harvested by centrifugation (10,000 × g, 5 min, 4°C) and resuspended in one-fifth of the initial volume of fresh Luria–Bertani (LB) medium. Bacteriophages were added with an optimal MOI and allowed to adsorb at 37°C with the rotary speed of 160 r/min for 10 min. The suspension was then centrifuged at 12,000 × g for 5 min, resuspended in 30 mL of LB broth, and serial dilutions of this suspension were carried out and incubated at 30°C. At regular intervals, aliquots (100 µL) of each dilution were collected for bacteriophage counts. The burst time and burst size were calculated from the one-step growth curve. Burst size was calculated as the ratio of the number of released phage progeny at the plateau to the initial number of host cells at the beginning of the latent period.

### Whole-genome sequencing and bioinformatics analysis

Whole-genome sequencing of vB3530 was done in a previous study (22). Snippy software was used to construct a core SNP phylogenetic tree (https://github.com/tseemann/snippy). FASTTREE V2.1.10 and iTOL were used to generate and visualize the phylogenetic tree (http://www.microbesonline.org/fasttree/, https://itol.embl.de/), respectively (47).

### Isolation of the bacteriophage-resistant strains

*P. aeruginosa* (1 × 10^7^ CFU/mL) was cultured to the logarithmic growth stage and then transferred to fresh LB liquid medium in test tubes. Bacteriophage vB3530 (2 × 10^7^ PFU/mL) was added, and no bacteriophage vB3530 was set as the control and incubated at 180 rpm, 37°C. OD_600_ was detected every 2 h and recorded for a total of 24 h. The following day, the aforementioned bacterial solution was diluted 10-fold and disseminated on LB agar plates and incubated at 37°C. Ten single colonies (bacteriophage-resistant strain TL3780-R) were selected and subcultured thrice on LB agar in the absence of the phage. The bacterial solution of *P. aeruginosa* TL3780 and bacteriophage-resistant strain TL3780-R with 200 µL logarithmic growth stage (OD_600_ = 0.6) were mixed with 8 mL semi-solid medium. After solidification, 5 µL of the phage preservation solution was added, and then the phage was naturally dried and then cultured in a constant temperature incubator at 37°C for 12 h to observe the appearance of plaque (50). The experiment was repeated three times.

### Sodium dodecyl sulfate polyacrylamide gel electrophoresis

An LPS extraction kit (iNtRON Biotechnology, Korea) was used following the manufacturer’s instruction. LPS was then separated on Ready Gel Precast Tris-HCl polyacrylamide gels with 15% and 5% acrylamide in the separating and stacking gels, respectively (Bio-Rad Laboratories, USA) in buffer with 2% SDS and fixed overnight in buffer with 10% acetic acid and 40% methanol. The gels were stained with a silver staining kit (Bio-Rad Laboratories, USA) following the manufacturer’s instruction. All solutions were prepared fresh before use. The experiment was repeated three times (51).

### Bacterial growth monitoring

To assess the growth of phage-resistant bacteria, we performed growth curve experiments. In short, the bacterial cultures were grown at 37°C for 24 h, followed by measurement of the absorbance at 600 nm every 2 h. Finally, the bacterial growth curves for bacteriophage-resistant strain TL3780-R (TL3780-R1 to TL3780-R10) and the parental strain TL3780 were determined by plotting the values against time.

### Biofilm-formation assay

*P. aeruginosa* cultures for bacteriophage-resistant strain TL3780-R (TL3780-R1 to TL3780-R10) and the parental strain TL3780 were cultured overnight on Columbia blood plates. The bacterial suspension was prepared and adjusted to 0.5 McFarland and diluted 1:100 in fresh LB broth. The suspension was then spread on 96-well plates and cultured overnight at 37°C for 24 h. The culture supernatant was discarded after incubation. The plates were washed thrice with water to remove any remaining planktonic cells. Biofilms formed on the plates were stained for 15 min with 1% crystal violet, the excess dye was removed by washing thrice with 1 × PBS, and the bound crystal violet was solubilized in 95% ethanol. The absorbances were measured at 600 nm (52).

### Motility assay

For the twitching assay, *P. aeruginosa* cultures for bacteriophage-resistant strain TL3780-R (TL3780-R1 to TL3780-R10) and the parental strain TL3780 were inoculated on the bottom of a petri dish. After incubation at 37°C for 24 h, the agar was gently removed, and the petri dish was air-dried. Then, 1% crystal violet solution was used to stain the plate agar interface for 10 min. Finally, the petri dish was rinsed, and the crystal violet-stained twitching pattern was evaluated. The migration distance around the incubation site was also measured. The migration distance was found to be directly proportional to the motility ability (52, 53).

### Pyocyanin assay

The pyocyanin production was measured as described previously (52). *P. aeruginosa* cultures for bacteriophage-resistant strain TL3780-R (TL3780-R1 to TL3780-R10) and the parental strain TL3780 were grown for 16–20 h. The pyocyanin concentration was estimated by vortexing 7.5 mL of the filtered supernatant with 4.5 mL of chloroform until the color turned to greenish blue. The samples were then centrifuged (10,000 × g for 10 min), and 3 mL of the resultant blue liquid was transferred into a fresh tube containing 1.5 mL of 0.2 M HCl and agitated until the blue color changed to pink. The absorbance of the pink layer was measured at 520 nm after it was transferred into a cuvette. Pyocyanin absorbance at OD_520_ was normalized by culture cell density OD_600_ (54).

### Quantitative reverse transcription PCR (qRT-PCR)

The expression levels of *P. aeruginosa* QS circuit genes were evaluated by RT-qPCR, as described previously (55). *P. aeruginosa* cultures for bacteriophage-resistant strain TL3780-R (TL3780-R1 and TL3780-R2) and the parental strain TL3780 were incubated in fresh LB broth at 37°C under 180 rpm until reaching the logarithmic growth phase (OD_600_ 0.5–0.6). Total RNA was extracted from planktonic bacteria using the RNeasy Mini Kit (Qiagen, Valencia, CA, USA) in accordance with the manufacturer’s instructions. Purified RNA was reverse transcribed onto cDNA using the cDNA Synthesis Kit (TaKaRa, Tokyo, Japan) in accordance with the manufacturer’s instructions. The gene expression levels were measured by qRT-PCR using the TB Green Premix Ex Taq II (Tli RNase H Plus) (2×) (Takara) with specific primers listed in Table S3. *rpsL* was used as an internal control to normalize the data. The gene expression levels were calculated using the 2^−△△^Ct method.

### Antimicrobial susceptibility testing

The MICs of antibacterial agents were determined by the microdilution broth method according to the guidelines recommended by the latest Clinical and Laboratory Standards Institute (CLSI, 2022). Briefly, an overnight cultured single colony was suspended in sterile NaCl (0.85%), and the suspensions were adjusted to the turbidity equal to 0.5 McFarland standard (1.5 × 10^8^ CFU/mL). Then, the mixture was further diluted to 1:100 and evenly added into the drug-containing 96-well microplate; the results were observed after incubation for 16–18 h at 37°C. *P. aeruginosa* ATCC 27853 was employed as the quality control strain. MIC values were determined by three independent experiments (56).

### Statistical analysis

The statistical significance of the differences between the control and experimental groups was evaluated by Student’s *t*-test. For all analyses: *P < 0.05, **P < 0.01, ***P < 0.001, ****P < 0.0001, and ns *P* > 0.05. All data analyses were performed using GraphPad Prism (version 8.0, GraphPad Software).

## Data Availability

The complete genome sequence of bacteriophage vB3530 was deposited in GenBank under the accession number OR075999.

## SUPPLEMENTAL MATERIAL

Table S1 msphere.00553-23-s0001.docx The sequence types (ST) and clinical characteristics of *P. aeruginosa*.

Table S2 msphere.00553-23-s0002.doc vB3530 phage gene function annotation.

Table S3 msphere.00553-23-s0003.doc Primers for RT-qPCR.

ASM does not own the copyrights to Supplemental Material that may be linked to, or accessed through, an article. The authors have granted ASM a non-exclusive, world-wide license to publish the Supplemental Material files. Please contact the corresponding author directly for reuse.
