# Supplementary material for: Resistance, mechanism, and fitness cost of specific bacteriophages for Pseudomonas aeruginosa
Source: mSphere. 2024 Feb 1;9(2):e00553-23. doi: 10.1128/msphere.00553-23 (PMC10900902; doi:10.1128/msphere.00553-23)
Supplement: Table S1 — The sequence types (ST) and clinical characteristics of P. aeruginosa. [file msphere.00553-23-s0001.docx]

| **Strain** | **ST** | **Sample** | **Host range** |
| --- | --- | --- | --- |
| TL3569 | ST381 | Sputum | + |
| TL3649 | ST856 | Urine | - |
| TL3651 | ST2449 | Sputum | + |
| TL3652 | ST1968 | Sputum | + |
| TL3670 | ST277 | Sputum | + |
| TL3674 | ST274 | Sputum | + |
| TL3683 | ST644 | Sputum | + |
| TL3692 | ST1968 | Drainage | + |
| TL3706 | ST644 | Sputum | + |
| TL3727 | ST1249 | Sputum | - |
| TL3733 | ST980 | Sputum | + |
| TL3761 | ST242 | Sputum | + |
| TL3763 | ST377 | Blood | - |
| TL3767 | ST377 | Purulent secretion | + |
| TL3780 | ST1682 | Urine | + |
| TL3783 | ST463 | Sputum | + |

Table S1. The sequence types (ST) and clinical characteristics of *P. aeruginosa*. Host range of vB3530 determined on 16 bacterial strains. Clear lysis zone (+) and not lysis zone (−).
