# Supplementary material for: Resistance, mechanism, and fitness cost of specific bacteriophages for Pseudomonas aeruginosa
Source: mSphere. 2024 Feb 1;9(2):e00553-23. doi: 10.1128/msphere.00553-23 (PMC10900902; doi:10.1128/msphere.00553-23)
Supplement: Table S2 — vB3530 phage gene function annotation. [file msphere.00553-23-s0002.doc]

TableS2 vB3530 phage gene function annotation

| Feature | Number |
| --- | --- |
| Phage capsid and scaffold | 2 |
| Phage terminase，large subunit | 1 |
| Phage protein | 27 |
| Phage minor capsid protein | 1 |
| Phage tail fiber | 3 |
| Phage internal protein | 1 |
| Phage baseplate | 1 |
| Phage endolysin | 1 |
| DNA ligase | 1 |
| Phage DNA-binding protein | 1 |
| Phage DNA helicase | 2 |
| DNA polymerase Ⅲalpha subunit | 1 |
| Phage DNA systhesis | 1 |
| Thymidylate synthase ThyX | 1 |
| Phage tail assembly protein | 1 |
| Phage-associated DNA primase | 1 |
| Hypothetical protein | 49 |
