# Supplementary material for: Resistance, mechanism, and fitness cost of specific bacteriophages for Pseudomonas aeruginosa
Source: mSphere. 2024 Feb 1;9(2):e00553-23. doi: 10.1128/msphere.00553-23 (PMC10900902; doi:10.1128/msphere.00553-23)
Supplement: Table S3 — Primers for RT-qPCR. [file msphere.00553-23-s0003.doc]

Table S3 Primers for RT-qPCR

| **gene** | **type** | **sequence** |
| --- | --- | --- |
| *lasI* | F | 5‘- GGCTGGGACGTTAGTGTCAT-3’ |
|  | R | 5‘-AAAACCTGGGCTTCAGGAGT-3’ |
| *lasR* | F | 5‘-ACGCTCAAGTGGAAAATTGG-3’ |
|  | R | 5‘-TCGTAGTCCTGGCTGTCCTT-3’ |
| *rhlI* | F | 5‘-AAGGACGTCTTCGCCTACCT-3’ |
|  | R | 5‘- GCAGGCTGGACCAGAATATC-3’ |
| *rhlR* | F | 5‘-CATCCGATGCTGATGTCCAACC-3’ |
|  | R | 5‘-ATGATGGCGATTTCCCCGGAAC-3’ |
| *rpsL* | F | 5‘-GCAACTATCAACCAGCTGGTG-3’ |
|  | R | 5‘-GCTGTGCTCTTGCAGGTTGTG-3’ |
| *pqsA* | F | 5‘-GACCGGCTGTATTCGATTC-3’ |
|  | R | 5‘-GCTGAACCAGGGAAAGAAC-3’ |
| *mexA* | F | 5‘-AACCCGAACAACGAGCTG-3’ |
|  | R | 5‘-ATGGCCTTCTGCTTGACG-3’ |
| *mexC* | F | 5‘-TGGTAATCATCACGAACTC-3’ |
|  | R | 5‘-GCAGCAGATAACTTCCTT-3’ |
| *mexE* | F | 5‘-GTCGTAACCATTAACTTCTG-3’ |
|  | R | 5‘-ATCGTGGATGATGAACAA-3’ |
| *mexX* | F | 5‘-TGTATTTAAAGTGATAATATGAGTC-3’ |
|  | R | 5‘-CTTAGCCCATGATTTAAAAACACC-3’ |
